# Can biomarkers of extracellular matrix remodelling and wound healing be used to identify high risk patients infected with SARS-CoV-2?: lessons learned from pulmonary fibrosis

**DOI:** 10.1186/s12931-020-01590-y

**Published:** 2021-02-05

**Authors:** D. J. Leeming, F. Genovese, J. M. B. Sand, D. G. K. Rasmussen, C. Christiansen, G. Jenkins, T. M. Maher, J. Vestbo, M. A. Karsdal

**Affiliations:** 1 Nordic Bioscience, Herlev, Denmark; 2 Division of Respiratory Medicine, University of Nottingham, Nottingham, UK; 3 Keck School of Medicine, University of Southern California, Los Angeles, USA; 4 National Heart and Lung Institute, Imperial College, London, UK; 5 Division of Infection Immunity and Respiratory Medicine, The University of Manchester and Manchester University NHS Foundation Trust, Manchester, England

## Abstract

Pulmonary fibrosis has been identified as a main factor leading to pulmonary dysfunction and poor quality of life in post-recovery Severe Acute Respiratory Syndrome (SARS) survivor’s consequent to SARS-Cov-2 infection. Thus there is an urgent medical need for identification of readily available biomarkers that in patients with SARS-Cov-2 infection are able to; (1) identify patients in most need of medical care prior to admittance to an intensive care unit (ICU), and; (2) identify patients post-infection at risk of developing persistent fibrosis of lungs with subsequent impaired quality of life and increased morbidity and mortality. An intense amount of research have focused on wound healing and Extracellular Matrix (ECM) remodelling of the lungs related to lung function decline in pulmonary fibrosis (PF). A range of non-invasive serological biomarkers, reflecting tissue remodelling, and fibrosis have been shown to predict risk of acute exacerbations, lung function decline and mortality in PF and other interstitial lung diseases (Sand et al. in Respir Res 19:82, 2018). We suggest that lessons learned from such PF studies of the pathological processes leading to lung function decline could be used to better identify patients infected with SARS-Co-V2 at most risk of acute deterioration or persistent fibrotic damage of the lung and could consequently be used to guide treatment decisions.

## Background on COVID-19

The COVID-19 pandemic, caused by the novel virus Severe Acute Respiratory Syndrome Coronavirus-2 (SARS-CoV-2), has reached pandemic status, causing severe impacts on health, major reductions in life quality due to widespread quarantines and social distancing and huge economic impacts, all of which affecting many millions of people worldwide. Updated information on COVID-19 disease is continuing to emerge, including from Wuhan in China from where the virus was first reported [1], as well as from Europe and United States [2]. These data include descriptions of symptoms in patients presenting with SARS-CoV-2 infection and subpopulations at high risk of developing severe pneumonia [1]. Common symptoms include fever, dry cough and dyspnea [3]. Risk factors for severe COVID-19 disease and potentially fatal outcomes include pre-existing comorbidities such as type I or II diabetes, cardiovascular disease, chronic kidney disease, malignancy and lung conditions like pulmonary fibrosis, chronic obstructive disease, uncontrolled asthma or being a smoker [4–6]. A large group of patients presenting with severe SARS-CoV-2 driven pneumonia require Intensive Care Unit (ICU) admission and ventilatory support. Some patients develop Acute Respiratory Distress Syndrome (ARDS), potentially demonstrating a cytokine storm involving interleukin (IL)-1, IL-6 and TNF-alpha. The combination of these complications has the potential to promote lung inflammation and fibrosis [7]. In a number of early case series, the fatality rate in COVID-19 patients developing ARDS was > 50% [8]. There is an urgent medical need for identification of simple biomarkers that in patients infected by SARS-CoV-2 may; (1) identify patients in most need of medical care prior to admittance to an ICU, and; (2) identify patients post-infection at risk of developing persistent fibrosis of lungs with subsequent impaired quality of life and increased morbidity and mortality.

## SARS-CoV-2 infection and its main potential pathway within the lung

The SARS-CoV-2 virus has been reported to use the angiotensin converting enzyme 2 (ACE2) receptor and the viral entry-associated protease TMPRSS2 for cellular entry in the upper airway. ACE2 and TMPRSS2 have been detected in nasal and bronchial epithelium by immunohistochemistry. Gene expression occurs in approximately 2% of type II alveolar epithelial cells (AECs) [9–11], although one study reported the absence of ACE2 in the upper airway [12]. Sungnak et al. [11] investigated the gene expression in multiple datasets and various tissues and found that ACE2 rather than TMPRSS2 may be the limiting factor for viral entry at the initial infection stage. Additionally, the study showed that ACE2 was expressed at higher levels in multiple epithelial cells across the airway, especially in nasal secretory and ciliated cells, with only low levels of expression in type II AECs in the parenchyma. The high expression of receptors in the upper airways seems to be associated with a fairly high viral transmissibility [11]. A study on COPD has shown that COPD patients have an increased ACE2 gene expression in the small airway epithelial cells as compared to healthy individuals [6], Additionally, ACE2 expression was associated with smoking status with current smokers expressing higher levels of ACE2 as compared with former and never smokers. A higher expression of ACE2 may explain why such populations are more susceptible to infection with SARS-CoV-2.

AECs are squamous cells with long cytoplasmic extensions that are adapted to carry out gas exchange [13–14]. They cover 95% of the alveolar surface and provide crucial barrier function. Type II AECs are cuboidal in shape, more numerous than type I AECs and are responsible for the secretion of pulmonary surfactant in the alveoli [15]. Pulmonary surfactant reduces surface tension, prevents alveolar collapse and has additional functions in the innate immunity of the lung. Furthermore, type II AECs are crucial for upholding lung function due to their role in maintenance and repair of the injured alveolar epithelium. Following damage to type I AECs, the type II AECs initiate reparative processes including hyperplasia and eventually differentiation into type I AECs. The infection of type II AECs with SARS-CoV-2 may influence lung regeneration capabilities and surfactant production/function and may even aid the virus replication, although how this occurs given that less than 2% of type 2 cells express ACE2 remains to be determined. Surfactant dysfunction plays a major role in ARDS [16]. One route for the loss of functional surfactant is associated with the wound healing processes where fibrinogen leaks into the alveolar space and is converted to fibrin as part of the coagulation cascade. Fibrin inhibits pulmonary surfactant and thus promotes alveolar collapse that may in turn lead to fibroblast activation and fibrosis development [17]. Surfactant therapy is currently under investigation for treatment of COVID-19.

## Potential consequences of SARS-CoV-2 infection to the lung extracellular matrix

PF has been identified as one of the main factors leading to pulmonary dysfunction and poor quality of life in post-recovery Severe Acute Respiratory Syndrome (SARS) survivors [18–20]. Existing evidence, although scanty, suggests that PF could be a major complication of COVID-19 disease [2, 20, 21]. At present, there are no suggestions for potential mechanisms leading to PF during COVID-19 progression, nevertheless it is well known that lung inflammation and sustained lung damage is key to promoting PF. Interestingly, reports on the clinical manifestations within the lungs of COVID-19 patients investigated by computed tomography (CT) are emerging in the literature [20]. Lung lesions have been reported in COVID-19 patients with moderate to severe disease either in the upper or lower lobes showing uneven density with ground glass opacity (GGO) accompanied by fibrosis [22]. Other CT investigations in COVID-19 patients supported these findings, showing GGO with fibrosis as well as thickened septa and vascular wall thickening [3, 8]. Histologically, diffuse alveolar damage (DAD) has been seen as one of the most characteristic findings in non-survivors of COVID-19 as well as of SARS. Furthermore, fibrosis was accompanied by exudation and thrombosis commonly observed in the lung microvasculature accompanying the DAD [23]. Microthrombosis was also found in extrapulmonary organs infected by SARS-CoV-2, which was less reported in SARS [24]. Finally, in a study of COVID-19 patients receiving intensive care, around 40% developed ARDS and around half of these patients died [1]. Those who developed ARDS presented with greater dyspnoea and had comorbidities such as hypertension and diabetes. In addition, subjects were older, had higher fever, and had organ and coagulation dysfunction with higher lactate dehydrogenase and D-dimer compared to patients that did not develop ARDS. Thus, in general it appears that uncontrolled inflammation, and wound healing as well as PF are a consequence of COVID-19, making it of great interest to evaluate systemic biomarkers of these pathological relevant processes including markers of wound healing and Extracelullar Matrix (ECM) remodelling as potential prognostic markers in COVID-19 patients.

## Markers of lung extracellular matrix remodelling used to evaluate pulmonary fibrosis

A growing body of research has been undertaken to understand changes which occur in ECM dynamics related physiological impairment and lung function decline in e.g. idiopathic pulmonary fibrosis (IPF) [25–28].

**Fig. 1:**
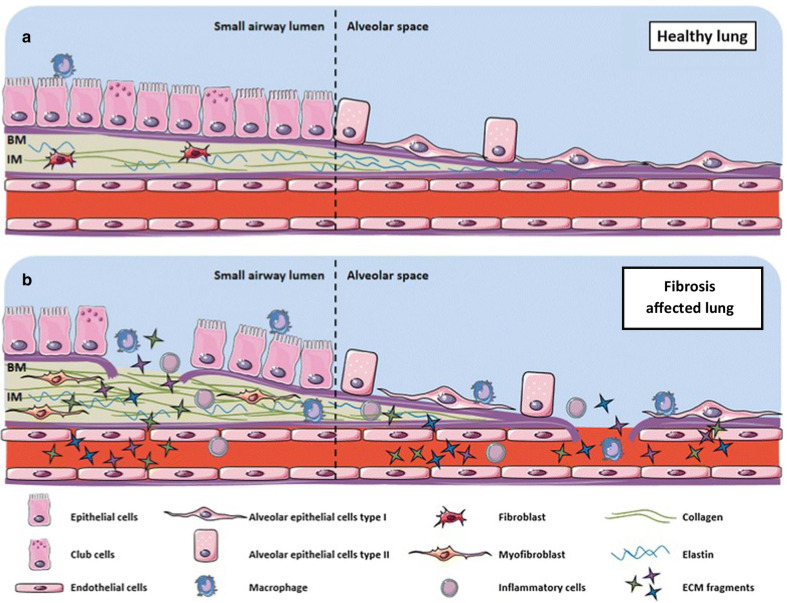
Extracellular matrix (ECM) composition in healthy (**a**) and fibrosis affected lungs (**b**). In healthy lungs, the epithelial cells create a tight barrier that blocks the entry of foreign particles from the inhaled air to the tissue. This is further enforced by the underlying basement membrane (BM) which mainly consists of type IV collagen. The interstitial matrix (IM) is placed below the BM and consists mainly of fibrillar collagens and elastin. In fibrosis affected lung, the continuous epithelial layer is disrupted, and the underlying BM is exposed. The inflammatory response to repeated tissue injury results in the up regulation of proteases and disruption of the BM, exposing the underlying IM to injury. In response to this, fibroblasts are activated and converted to pro-fibrotic myofibroblasts that secrete collagens which accumulate in the IM of the airway wall. Both collagens and elastin undergo proteolytic degradation in the airway and alveolar walls. The processes of synthesis and degradation release ECM fragments which may enter the bloodstream where they can be detected as biomarkers of ECM remodelling. Reproduced with slight modification from [30]

The lungs have a highly specialized ECM (Fig. 1). In healthy state the ECM forms a thin basement membrane layer in the small airways, separating the capillaries from the alveolar space, and allowing unimpeded gas diffusion (Fig. 1a). During fibrosis the ECM expands limiting this diffusion (Fig. 1b). The fibrotic ECM consists mainly of fibronectin and type I, III and VI collagens. Very simplistically, in lung fibrosis, the specialised basement membrane composed of an open structed type IV collagen backbone and laminin, permissive of diffusion, is replaced by a dense interstitial ECM consisting of a completely different set of collagens with, consequently, a different functionality [29].

The long-term effects of SARS-CoV-2 infection on the pulmonary system must be understood to better support future COVID-19 patients. An important research area to prevent the development of fibrotic consequences of COVID-19 is to understand the effects of the SARS-CoV-2 infection in to the lung ECM [29]. Data described here support that PF may be a complication of COVID-19, thus it may be of great interest to evaluate the ECM remodelling in patients with COVID-19 using markers of ECM remodelling. Such markers may potentially be utilized to evaluate severity of disease as well as predict clinical outcome, thus used as prognostic markers in patients known to be infected by SARS-CoV-2. The neoepitope technology for the systemic assessment of ECM turnover as a measure of organ fibrosis has previously been described [25, 26]. This technology utilizes monoclonal antibodies to detect the generation of newly formed epitopes of collagens and other ECM proteins released during inflammation and fibrosis progression as well as markers of wound healing that can reflect a disturbed coagulation [25, 31, 36]. The neoepitope technology allows for the detection of both fibrogenesis [32] and fibrolysis of up to twenty-eight different collagens, each contributing to the integrity and quality of the ECM in different organs, including the lungs [26]. These neoepitope markers have shown to be associated to PF progression and mortality in patients with IPF [25, 26] and systemic sclerosis (SSc) also known to present with pulmonary fibrosis [33]. Neoepitope markers of both fibrogenesis and fibrolysis were elevated in patients with IPF compared with healthy controls, and the change of these markers over a short period was significantly related to loss in lung function and increased mortality risk in IPF patients [25, 26]. Furthermore, a versican neoepitope markerhas been reported to become elevated during acute exacerbations of interstitial pulmonary fibrosis [34].

Key data on neoepitope biomarkers within PF are summarized in Table 1, to emphasize which tools are available that may be repurposed for COVID-19.

**Table 1:** Selected biomarkers of ECM and wound healing that in peer review publications have been shown to be associated with key features of PF and potentially may also be used for segregation of COVID-19 patients

Biomarker	Biological meaning	Prediction of delta FVC (IPF)	Prediction of death in IPF	References
C1M	MMP degraded type I collagen—type I collagen is the most abundant protein in the body, and MMPs are produced by inflammatory cells degrading the tissue resulting in C1M	yes	Yes	[25], 1
C3M	MMP degraded type III collagen—type III collagen is abundant in the interstitial ECM, and MMPs are produced by inflammatory cells degrading the tissue resulting in C3M	yes	Yes	[25]
C6M	MMP degraded type VI collagen—type VI collagen is very abundant in lung ECM, and MMPs are produced by inflammatory cells degrading the tissue resulting in C6M	yes	Yes	[25, 26]
PRO-C6	Type VI collagen formation. Also known as the collagen hormone Endotrophin	yes	No	[26]
PRO-C3	Type III collagen formation, one of the most upregulated collagens in fibrotic tissues	yes	Yes	[26]
CRPM	MMP degradaded C-reactive protein a marker of local inflammaion	Yes	Yes	[25, 26]

**Fig. 2:**
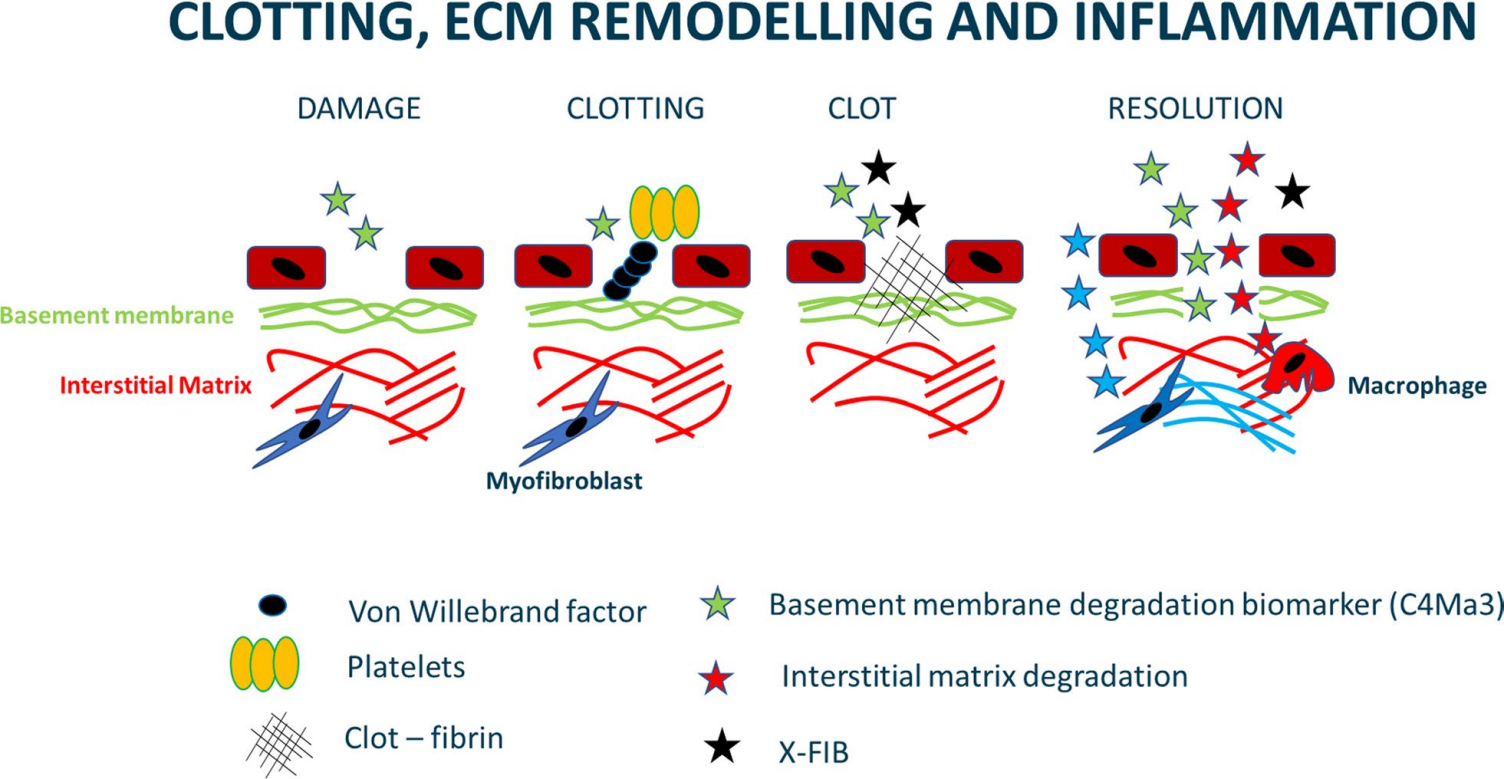
Schematic drawing of the basement membrane (in green) underlying the lung epithelium and the interstitial matrix (in red), supporting the tissue. After cellular damage the epithelium is compromised, clotting occurs which results in remodelling of the tissue. When overactivated, fibroblasts (blue) produce fibrillar collagen limiting oxygen diffusion. During these processes, small fragments of proteins related to these molecular events are released into the circulation, where they may be used as biomarkers

Neoepitope markers of wound healing should also be considered as valuable markers in COVID19 patients and are under investigation in PF at the moment. As eluted to earlier, it has been found that D-Dimer is elevated in ARDS patients compared to non-ARDS patients and indicating increased woundhealing during ARDS. The relationship between cell damage and the resulting wound healing and ECM remodelling is schematically represented in Fig. 2. In the case that fibroblasts are overactivated, they initiate production of fibrillar collagens (type I, III, V collagen), which are not permeable for oxygen, unlike the networking collagens of the basement membrane (type IV and VIII collagen) [29, 31].

## Routine clinical parameters as markers of outcome in COVID-19

Several groups have investigated the use of routine clinical parameters assessed in patients infected by SARS-CoV-2. Interestingly, a machine learning approach was used upon 398 patients by Booth et al. [35] with the aim to develop an algorithm to predict outcome; developing one which was able to predict outcome in patients with COVID-19, with a 91% sensitivity and 91% specificity for patient expiration up to 48hrs prior to the event. The model included C-reactive protein (CRP), blood urea nitrogen, serum calcium, serum albumin and lactic acid. Potentially, markers of ECM remodelling, as described here may be used to predict outcome, event at an earlier time point, nevertheless this is speculations. Furthermore, the kinetics of routine clinical markers was followed in fifty patients diagnosed with COVID-19, and admitted to the hospital [36]. Patients were followed during hospitalization and one month prior to discharged, as well as stratified into non-ICU and ICU severity. Mapping of blood parameters showed increased CRP, ferritin, lactate dehydrogenase, white blood cell count at admission, Furthermore, differences in levels of CRP, ferritin, D-Dimer, fibrinogen, lymphocyte count, and neutrophil count were different in non-ICU vs ICU patients, and kinetics showed that peak in such markers also differed between early versus later time points. Authors concluded that high levels of CRP, D-dimer, ferritin, lymphopenia and eosinopenia proved to be beneficial markers for risk stratification. Finally, markers of inflammatory and endothelial dysfunction were evaluated for their accuracy to predict a 30-day intubation or mortality as well as oxygen requirement in 76 COVID-19 patients [37]. The study showed that a soluble triggering receptor expressed on myeloid cells provided the best prognostic accuracy with an area on the receiver-operating curve of 0.86, with IL-6 providing the next best accuracy of 0.84.

## Conclusion

In conclusion, there are important lessons learned from diseases leading to PF in which key ECM remodelling and wound healing biomarkers are predicting exacerbations of disease and death.These important data and key learnings have the potential to be repositioned for COVID-19 research. Potentially, systemic markers of ECM turnover, inflammation and wound healing, the latter based on data from previous publications, may provide value in the clinical evaluation of COVID-19 patients, with the aim of assisting in the prediction of prognosis during the acute phase of disease and could potentially identify COVID-19 survivors at risk of developing permanent pulmonary damage and fibrosis.

## Data Availability

Not applicable.

## Abbreviations

ACE2: Angiotensin converting enzyme 2
AEC: Type II alveolar epithelial cells
AST: Aspartate transaminase
ALT: Alanine transaminase
ARDS: Acute respiratory distress syndrome
BM: Basement membrane
COPD: Chronic obstructive pulmonary disease
CT: Computed tomography
DAD: Diffuse alveolar damage
ECM: Extracellular matrix
ICU: Intensive care unit
IL: Interleukin
IM: Interstital matrix
IPF: Idiopathic pulmonary fibrosis
GGO: Ground glass opacity
PF: Pulmonary fibrosis
SARS: Severe acute respiratory syndrome
SARS-CoV-2: Severe acute respiratory syndrome coronavirus-2
SSC: Systemic sclerosis

## References

[1] Wu C (2020). Risk factors associated with acute respiratory distress syndrome and death in patients with coronavirus disease 2019 pneumonia in Wuhan, China. JAMA Intern Med.

[2] Spagnolo P (2020). Pulmonary fibrosis secondary to COVID-19: a call to arms?. Lancet Respir Med.

[3] Rodriguez-Morales AJ (2020). Clinical, laboratory and imaging features of COVID-19: A systematic review and meta-analysis. Travel Med. Infect. Dis..

[4] Du Y (2020). Clinical features of 85 fatal cases of COVID-19 from Wuhan: a retrospective observational study. Am J Respir Crit Care Med.

[5] Certain Medical Conditions and Risk for Severe COVID-19 Illness | CDC. https://www.cdc.gov/coronavirus/2019-ncov/need-extra-precautions/people-with-medical-conditions.html. Accessed 12 Nov 2020

[6] Leung JM (2020). ACE-2 expression in the small airway epithelia of smokers and COPD patients: implications for COVID-19. Eur Respir J.

[7] Conti P (2020). Induction of pro-inflammatory cytokines (IL-1 and IL-6) and lung inflammation by Coronavirus-19 (COVI-19 or SARS-CoV-2): anti-inflammatory strategies. J Biol Regul Homeost Agents.

[8] Wu J (2020). Novel coronavirus pneumonia (COVID-19) CT distribution and sign features. Zhonghua Jie He He Hu Xi Za Zhi.

[9] Qi F, Qian S, Zhang S, Zhang Z (2020). Single cell RNA sequencing of 13 human tissues identify cell types and receptors of human coronaviruses. Biochem Biophys Res Commun.

[10] Zou X (2020). Single-cell RNA-seq data analysis on the receptor ACE2 expression reveals the potential risk of different human organs vulnerable to 2019-nCoV infection. Front Med.

[11] Sungnak W (2020). SARS-CoV-2 entry factors are highly expressed in nasal epithelial cells together with innate immune genes. Nat Med.

[12] Hamming I (2004). Tissue distribution of ACE2 protein, the functional receptor for SARS coronavirus. A first step in understanding SARS pathogenesis. J. Pathol..

[13] Saladin KS. Human anatomy. Fith Edition, NY: McGraw Hill Education; 2013. ISBN13:9780073403700

[14] Weinberger SE, Cockrill BA, Mandel J (2018). Principles of pulmonary medicine. Principles of pulmonary medicine.

[15] Fehrenbach H (2001). Alveolar epithelial type II cell: defender of the alveolus revisited. Respir Res.

[16] Anzueto A (2002). Exogenous surfactant in acute respiratory distress syndrome: more is better. Eur Respir J.

[17] Günther A (2001). Surfactant alteration and replacement in acute respiratory distress syndrome. Respir Res.

[18] Ooi GC (2004). Severe acute respiratory syndrome: temporal lung changes at thin-section CT in 30 patients. Radiology.

[19] Zhang P (2020). Long-term bone and lung consequences associated with hospital-acquired severe acute respiratory syndrome: a 15-year follow-up from a prospective cohort study. Bone Res..

[20] Wang J (2020). Advances in the research of mechanism of pulmonary fibrosis induced by Corona Virus Disease 2019 and the corresponding therapeutic measures. Zhonghua Shao Shang Za Zhi.

[21] Guan W (2020). Clinical characteristics of coronavirus disease 2019 in China. N Engl J Med.

[22] Xu Y-H (2020). Clinical and computed tomographic imaging features of novel coronavirus pneumonia caused by SARS-CoV-2. J Infect.

[23] Zhang T, Sun LX, Feng RE (2020). Comparison of clinical and pathological features between severe acute respiratory syndrome and coronavirus disease 2019. Zhonghua Jie He He Hu Xi Za Zhi.

[24] Klok FA (2020). Incidence of thrombotic complications in critically ill ICU patients with COVID-19. Thromb Res.

[25] Jenkins RG (2015). Longitudinal change in collagen degradation biomarkers in idiopathic pulmonary fibrosis: an analysis from the prospective, multicentre PROFILE study. Lancet Respir Med.

[26] Organ LA (2019). Biomarkers of collagen synthesis predict progression in the PROFILE idiopathic pulmonary fibrosis cohort. Respir Res.

[27] Kristensen JH (2015). Levels of circulating MMP-7 degraded elastin are elevated in pulmonary disorders. Clin Biochem.

[28] Leeming DJ (2012). Serological investigation of the collagen degradation profile of patients with chronic obstructive pulmonary disease or idiopathic pulmonary fibrosis. Biomark Insights.

[29] Karsdal MA (2017). The good and the bad collagens of fibrosis—their role in signaling and organ function. Adv Drug Deliv Rev.

[30] Sand JMB (2016). High levels of biomarkers of collagen remodeling are associated with increased mortality in COPD—results from the ECLIPSE study. Respir Res.

[31] Karsdal MA (2020). Collagen biology and non-invasive biomarkers of liver fibrosis. Liver Int.

[32] Karsdal MA (2013). Extracellular matrix remodeling: the common denominator in connective tissue diseases. Possibilities for evaluation and current understanding of the matrix as more than a passive architecture, but a key player in tissue failure. Assay Drug Dev Technol.

[33] Juhl P (2018). Serum biomarkers of collagen turnover as potential diagnostic tools in diffuse systemic sclerosis: a cross-sectional study. PLoS ONE.

[34] Sand JMB (2018). A serological biomarker of versican degradation is associated with mortality following acute exacerbations of idiopathic interstitial pneumonia. Respir. Res..

[35] Booth AL, Abels E, McCaffrey P (2020). Development of a prognostic model for mortality in COVID-19 infection using machine learning. Mod Pathol.

[36] Khourssaji M (2020). A biological profile for diagnosis and outcome of COVID-19 patients. Clin Chem Lab Med.

[37] Van Singer M (2020). COVID-19 risk stratification algorithms based on sTREM-1 and IL-6 in emergency department. J Allergy Clin Immunol.

